# Anti-inflammatory effects of miRNA-146a induced in adipose and periodontal tissues

**DOI:** 10.1016/j.bbrep.2020.100757

**Published:** 2020-04-21

**Authors:** Taiki Sanada, Tomomi Sano, Yusuke Sotomaru, Rehab Alshargabi, Yosuke Yamawaki, Akiko Yamashita, Hiroaki Matsunaga, Misaki Iwashita, Takanori Shinjo, Takashi Kanematsu, Tomoichiro Asano, Fusanori Nishimura

**Affiliations:** a Section of Periodontology, Kyushu University Faculty of Dental Science, Fukuoka, Japan; b Natural Science Center for Basic Research and Development, Hiroshima University, Hiroshima, Japan; c Department of Advanced Pharmacology, Daiichi University of Pharmacy, Fukuoka, Japan; d Laboratory of Cell Biology and Pharmacology, Kyushu University Faculty of Dental Science, Fukuoka, Japan; e Department of Biological Chemistry, Hiroshima University Institute of Biomedical and Health Sciences, Hiroshima, Japan

**Keywords:** MicroRNA-146a, Inflammation, Cytokine, Obesity, Periodontal disease

## Abstract

MicroRNA (miRNA) plays an important role in diverse cellular biological processes such as inflammatory response, differentiation and proliferation, and carcinogenesis. miR-146a has been suggested as a negative regulator of the inflammatory reaction. Although, it has been reported as expressed in inflamed adipose and periodontal tissues, however, miR-146a's inhibitory effects against inflammatory response in both the tissues, are not well understood. Therefore, in this study, the inhibitory effects of miR-146a on both adipose and periodontal inflammation, was investigated. *In vitro* study has revealed that miR-146a transfection into either adipocytes or gingival fibroblasts, has resulted in a reduced cytokine gene expression, observed on co-culturing the cells with macrophages in the presence of lipopolysaccharides (LPS), in comparison to the control miRNA transfected. Similarly, miR-146a transfection into macrophages resulted in a reduced expression of TNF-α gene and protein in response to LPS stimulation. *In vivo* study revealed that a continuous intravenous miR-146a administration into mice via tail vein, protected the mice from developing high-fat diet-induced obesity and the inflammatory cytokine gene expression was down-regulated in both adipose and periodontal tissues. miR-146a appeared to be induced by macrophage-derived inflammatory signals such as TNF-α by negative feed-back mechanism, and it suppressed inflammatory reaction in both adipose and periodontal tissues. Therefore, miR-146a could be suggested as a potential therapeutic molecule and as a common inflammatory regulator for both obesity-induced diabetes and related periodontal diseases.

## Highlights

• miR-146a induced by adipose and periodontal inflammation via macrophage mediators.
• miR-146a is a negative inflammation regulator in adipose and periodontal tissues.
• miR-146a is a potential therapeutic target against obesity and periodontal diseases.

## 1 Introduction

Obesity is a multifactorial chronic disorder, wherein, disease onset is greatly influenced by genetic and environmental factors [1]. It further up-regulates the risk of developing various life-threatening chronic diseases such as cancer and coronary heart diseases. Obesity appears to influence the systemic energy metabolism and inflammatory responses, evokes low-grade inflammatory status throughout the body, and exacerbates periodontal inflammation as well [2]. Human and animal studies have suggested a close link between obesity and periodontal diseases [3,4]. Adipose tissue-derived inflammatory response such as increased tumor necrosis factor-α (TNF-α) production is suggested to exacerbate the systemic low-grade inflammation, which further act as a deteriorating factor for other inflammatory diseases such as periodontal disease and inflammation. However, when up-regulated, it is suggested to, in turn influence adipose tissue inflammation which may further lower insulin sensitivity [5].

MicroRNA (miRNA), a single-strand, short RNA with 21–25 nucleotides, plays an important role in eukaryotic post-transcriptional mRNA stability. Therefore, it regulates several fundamental biological processes such as cell development, differentiation, proliferation, and cell death. In mammalian cells, more than 2,500 miRNAs have been described to regulate approximately 30–90% of the gene expression [6]. Interestingly, some miRNAs have been reported to be specifically expressed in inflammatory reactions [7], suggesting that such miRNAs play important roles in inflammatory responses.

Previously, miRNA expression in healthy and inflamed gingival tissues has been compared with respect to the interactions between periodontal disease and miRNA expression [8,9]. Additionally, expression of several miRNAs has been reported as up-regulated in the inflamed gingival tissues of obese subjects when compared to those of systemically healthy subjects [10]. Under inflammatory conditions, miRNA-146a (miR-146a) has been reported to be highly expressed in the white adipose tissues, whereas, miR-146a transfection into white adipocytes, has demonstrated down-regulation of the inflammatory cytokine expression [11]. Furthermore, a positive correlation was observed between gingival miR-146a expression and periodontal clinical parameter as assessed by periodontal pocket depth [12]. Although, miR-146a is suggested to be a negative regulator of NF-κB inflammatory signaling cascade [13], its precise anti-inflammatory effect in both adipose and periodontal tissues is not well understood. Furthermore, the chronic effects of its long-term administration into animal model is not performed yet. Therefore, in this study, the anti-inflammatory effects of miR-146a *in vitro* and *in vivo*, was accessed.

## 2 Materials and methods

### 2.1 Cells and cell culture

Murine 3T3-L1 preadipocytes (American Type Culture Collection, VA, USA) were maintained in Dulbecco's modified Eagles medium (DMEM) (Nacalai Tesque, Japan) containing 10% calf serum in an atmosphere of 5% CO_2_ at 37 °C. Two days after the 3T3-L1 fibroblasts had reached confluence, differentiation was induced by treating the cells with DMEM containing 4 μg/ml dexamethasone (Sigma Aldrich, MO, USA), 0.5 mM 3-isobutyl-1-methylxanthine (Sigma Aldrich, MO, USA), 200 nM insulin (Cell Science & Technology Institute, Japan) and 10% fetal bovine serum (FBS) (Biowest, France) for 48 h. Cells were fed with DMEM containing 10% FBS and 1 μM insulin every other day. Murine gingival fibroblast ESK-1 which were kindly gifted from professor John R. Klein (Department of Diagnostic Sciences, Dental Branch, University of Texas Health Science Center at Houston, TX, USA). ESK-1 cells were maintained in DMEM containing 10% FBS. Murine macrophage RAW264.7 cells were maintained in DMEM containing 10% FBS. Co-culture of adipocytes or gingival fibroblasts and macrophages was performed using a transwell system (Greiner, Austria) with a 0.4 μm porous membrane to separate the upper and lower chambers. 5 × 10^4^ differentiated 3T3-L1 cells or ESK-1 cells were cultured in the lower chamber, while 1 × 10^5^ RAW264.7 cells were cultured in the upper chamber. As *in vitro* models of inflamed adipose or gingival tissues, 3T3-L1 adipocytes or ESK-1 gingival fibroblasts were stimulated with LPS-stimulated macrophage-derived culture medium. RAW264.7 macrophages were stimulated with 1 ng/ml LPS for 24 h, and the culture medium was collected and added to each cell culture. *Escherichia coli* LPS (Sigma Aldrich, MO, USA) was used for the experiments.

### 2.2 miRNA microarray analysis

miRNA microarray analysis was performed in adipocytes co-cultured with macrophages in the presence or absence of LPS. All procedure is essentially the same as our mRNA microarray analysis as described previously [14]. Microarray and related cluster analysis were performed by Cosmo bio, Inc.

### 2.3 Real-time PCR analysis

Total RNA was extracted from the indicated cells or tissues using ISOGEN-II (NIPPON GENE, Japan) and reverse transcribed using Prime Script RT reagent Kits (Takara, Japan). RT-PCR was performed using the KAPA SYBR FAST qPCR Kits (Kapa Biosystems, MA, USA) and Step One Plus Real Time PCR System (Applied Biosystems, CA, USA). Relative mRNA genes were normalized to the *Gapdh* mRNA level and relative expression levels were determined by the comparative Ct method. Primer sequences are shown in Supplemental Table 1. For miRNA quantification, total RNA was reverse transcribed using the miScript II RT Kit (Qiagen, Germany) and analyzed by real-time PCR using the miScript SYBR Green PCR Kit and the miScript primer assay for miR-146a (Qiagen, Germany). Results were normalized to RNU6B (Qiagen, Germany).

### 2.4 Western immunoblot assay

The cells were solubilized with CytoBuster Protein Extraction Reagent (Millipore, MA, USA). Equal amounts of protein from whole cell lysates were resolved by SDS-PAGE. The proteins were then transferred to polyvinylidene difluoride membranes (Millipore, MA, USA) using the semi-dry system (Bio-Rad Laboratories, CA, USA). Primary antibodies were as follows: anti-mouse Gapdh and Jnk, pJnk (Cell Signaling Technology, MA, USA), Irak1 (GeneTex, CA, USA), Traf6 (Santa Cruz Biotechnology, CA, USA). After incubation with secondary antibodies (HRP-conjugated anti-rabbit and anti-mouse, Cell Signaling Technology, MA, USA), immunoreactive proteins were visualized using enhanced chemiluminescence (Chemi-Lumi One Super, Nacalai Tesque, Japan), and signals were analyzed using Image Quant LAS4000 (GE Healthcare, UK).

### 2.5 Cytokine assay

The cells were stimulated with LPS for 24 h, and culture supernatant was collected. Tnf-α in the culture supernatants were measured by commercial ELISA kit (mouse Tnf-α enzyme-linked immunosorbent assay kits, R&D Systems, MN, USA).

### 2.6 Transfection of miRNA mimics

3T3-L1, ESK-1 or RAW264.7 cells were transfected with 20 nM miR-146a mimic (*Syn*-mmu-miR-146a-5p, Qiagen, Germany) or Negative control (Negative Control cel-miR-39-3p, Qiagen, Germany), and 0.32 μl/cm^2^ Lipofectamine RNAiMAX for 3T3-L1, ESK-1 and 0.64 μl/cm^2^ for RAW264.7 (Invitrogen, Germany) were used as transfection reagents. Cells were forward transfected for 24 h according to the manufacture's protocol.

### 2.7 Animals

C57BL/6 mice were fed a normal diet (ND) with 5% fat (MF: Oriental Yeast Co, Ltd., Japan), or high fat diet (HFD) containing 60% fat per calorie (HFD-60: Oriental Yeast Co. Ltd. Japan). For obesity experiments, HFD feeding was started at 4 weeks after birth. Adipose tissues and gingival tissues were obtained at 12 weeks after sacrificing the mice.

### 2.8 *In vivo* miR-146a injection study

C57BL/6 male mice at 4 weeks of age (n = 5 in each group) were divided into two groups: HFD mice injected with miR-146a and HFD mice injected with control miRNA. A chemically modified HPLC purified miR-146a duplex and miRNA negative control duplex were purchased from Cosmo Bio (Japan). All oligos were dissolved in atelocollagen (Atelo gene, Koken Co., Japan) and injected via tail vein (200 μl) once a week for a period of 9 weeks at a dose of 4 nM/mouse. After 3 days of the final injection, all mice were sacrificed, and gingival and adipose tissue were obtained.

### 2.9 Histological analysis

Tissue samples were fixed in 4% paraformaldehyde solution (Nacalai Tesque, Japan). The paraffin-embedded sections were stained with hematoxylin and eosin. Adipocyte sizes were measured for two animals per group using a microscope (BZ-9000, Keyence BZ-X Analyzer). Average adipocyte size was calculated as described before [21].

### 2.10 Statistical analysis

All data are expressed as means ± SD. Statistical analyses were performed using Student's *t*-test or Tukey's test. Values of p < 0.05 were considered significant.

## 3 Results

### 3.1 Macrophage plays a crucial role in miR-146a induction. miR-146a transfection suppresses inflammatory gene expression in adipocytes, gingival fibroblasts, and macrophages

**Fig. 1:**
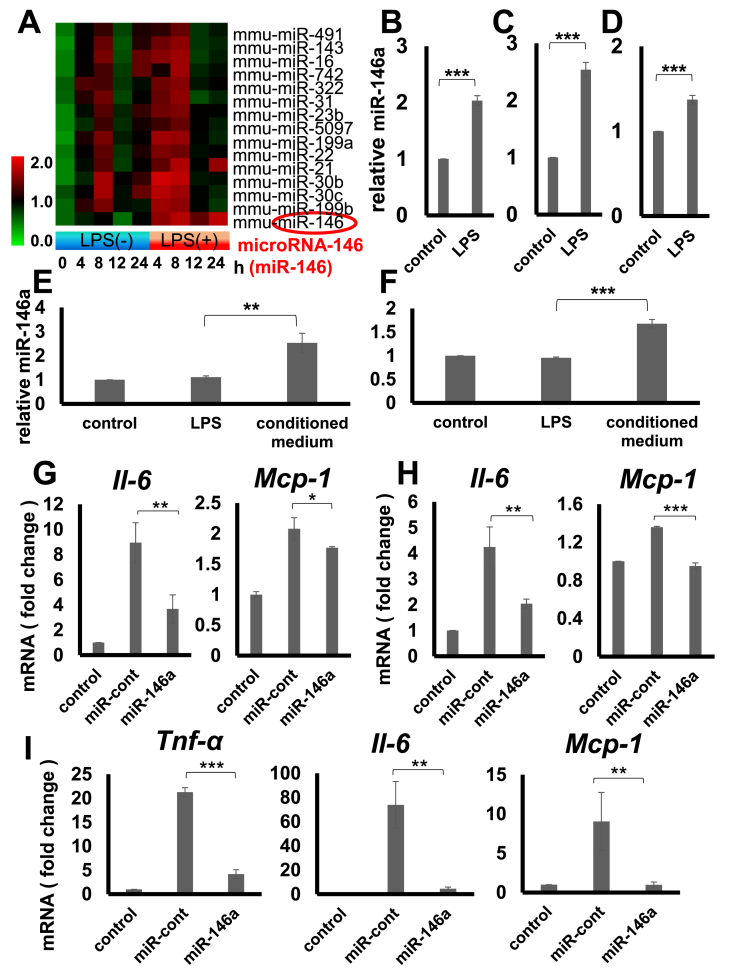
***In vitro* expression of miR-146a under inflammatory conditions. The effects of miR-146a transfection on inflammatory gene expression in adipocytes, gingival fibroblasts, and macrophages.** A) Expression of miR-146 in 3T3-L1 adipocytes co-cultured with macrophages with or without LPS stimulation determined by miRNA microarray. B–I) Real-time PCR analysis. B, C) Expression of miR-146a in 3T3-L1 adipocytes(B), ESK-1 cells(C) co-cultured with macrophages with or without LPS stimulation. D) Expression of miR-146a in macrophages with or without LPS stimulation. E, F) Expression of miR-146a in 3T3-L1 adipocytes(E), ESK-1 cells(F) cultured with culture supernatant of macrophages with or without LPS stimulation. G, H) Expression of *Il-6* and *Mcp-1* in miR-146a transfected 3T3-L1 adipocytes(G), ESK-1 cells(H) co-cultured with macrophages with or without LPS stimulation. I) Expression of *Tnf-α*, *Il-6*, and *Mcp-1* in miR-146a transfected macrophages with or without LPS stimulation. (B–D) ***P < 0.001 (n = 3), Student's *t*-test. (E–I) *P < 0.05, **P < 0.01, ***P < 0.001 (n = 3), Tukey's test.

Firstly, miRNA microarray analysis was performed in adipocytes when the cells were co-cultured with macrophages in the presence or absence of lipopolysaccharides (LPS). The results indicated highly induced miR-146 expression when the cells were co-cultured with macrophages and were LPS stimulated; and that the expression level was higher more than 2 times at 4, 8, 12, and 24 h following LPS stimulation as compared to the cells co-cultured with non-stimulated macrophages (Fig. 1A). Thereafter, the results were confirmed with reverse transcription-polymerase chain reaction. The miR-146a expression in gingival fibroblasts co-cultured with macrophages, with or without LPS stimulation, was further looked into in a similar way as the miRNA expression in a single culture of macrophages with or without LPS stimulation. The results indicated that LPS stimulation for 24 h, resulted in significantly increased miR-146a expression in the adipocytes (Fig. 1B), gingival fibroblasts (Fig. 1C), and macrophages (Fig. 1D). When the culture supernatant of macrophages, stimulated with LPS for 24 h, was used for adipocytes or gingival fibroblasts culture, miRNA-146a expression was noted to be induced in these cells as well (Fig. 1E and F). Interestingly, direct adipocytes and gingival fibroblast stimulation with LPS did not induce an increase in miR-146a expression in either of the cells, indicating that LPS-stimulated macrophage-derived supernatant contents, plays critical roles in miR-146a induction in both adipocytes and fibroblasts. All the analyses were normalized with *RNU6B*, a house-keeping gene.

Synthetic miR-146a mimic oligos or control oligos were transfected, which did not exhibit any homology with the reported mice genes. Following 24 h of transfection, the procedure was verified with a polymerase chain reaction (Fig. S1A). When the cells were co-cultured with macrophages with or without LPS stimulation, *Il-6* and *Mcp-1* expressions in miR-146a mimic-transfected adipocytes were significantly down-regulated as compared to the control miRNA-transfected cells (Fig. 1G). Similar results, transfection verification (Fig. S1B), and *Il-6* and *Mcp-1* down-regulation at identical time interval, was observed on replacing adipocytes with gingival fibroblasts (Fig. 1H). Furthermore, mimic or control transfection was performed in macrophages and its verification was performed after 36 h of transfection (Fig. S1C). On stimulating the cells with LPS, *Tnf-α*, *Il-6*, and *Mcp-1* gene expression was significantly down-regulated in mimic transfection group than the control transfection group (Fig. 1I).

### 3.2 miR-146a transfection into macrophages results in decreased phosphorylation of c-Jun N-terminal kinase (Jnk)

**Fig. 2:**
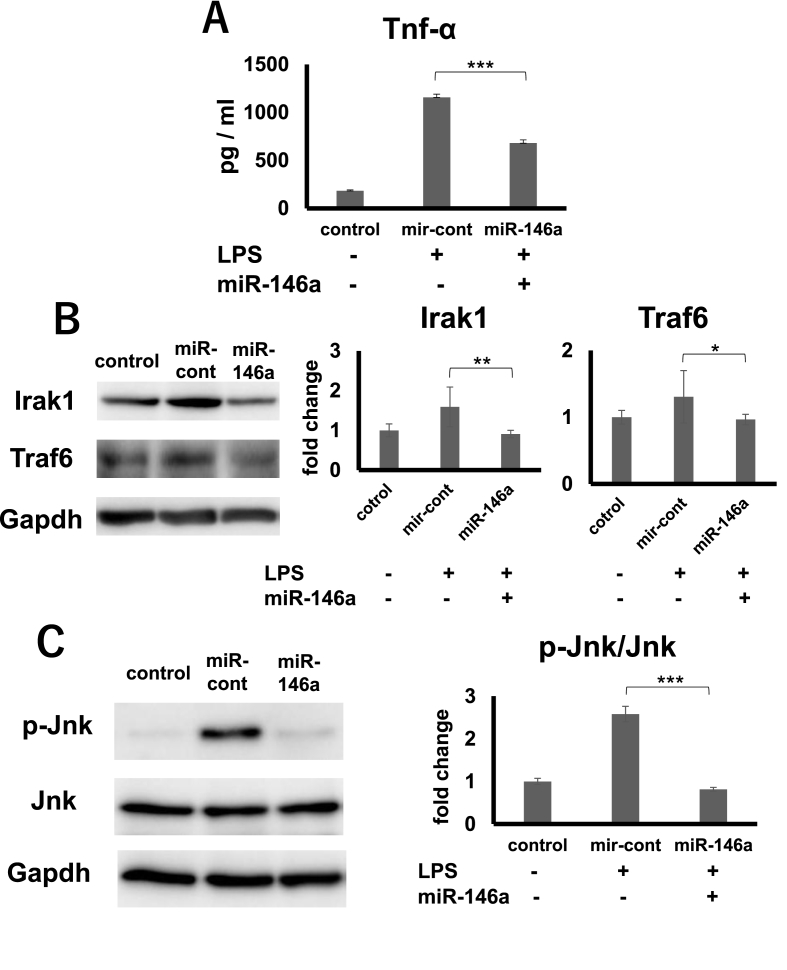
**The effects of miR-146a transfection on inflammatory gene cascades.** A) Tnf-α production from macrophages transfected with miR-146a mimic and its control with LPS stimulation. B) Irak1 and Traf6 expression in macrophages transfected with miR-146a mimic and its control with LPS stimulation. C) Jnk phosphorylation in macrophages transfected with miR-146a mimic and its control with LPS stimulation. D) Quantitation of Jnk phosphorylation by band intensity. *P < 0.05, **P < 0.01, ***P < 0.001 (n = 3), Tukey's test.

The macrophages were transfected with either mimic or control for 36 h, and later stimulated with LPS. TNF-α production after 4 h of LPS stimulation was significantly lowered in the mimic-transfected cells than control-transfected cells (Fig. 2A). Following a mimic or control transfection, the cells were also LPS-stimulated, and the cellular proteins were recovered 45 min after LPS stimulation. Interleukin-1 receptor-associated kinase 1 (Irak1) and TNF receptor-associated factor 6 (Traf6) protein expressions were significantly down-regulated in the mimic-transfected cells as well (Fig. 2B). Furthermore, following 90 min of LPS stimulation, Jnk phosphorylation was significantly down-regulated in the mimic-transfected cells (Fig. 2C). Usually, TLR4-mediated JNK phosphorylation occurs within 30 min after LPS stimulation. Therefore, this down-regulation was speculated as a result of second hit caused by autocrine TNF-α after LPS stimulation, as TNF-α production usually starts after 90 min of LPS stimulation.

### 3.3 miR-146a expression is up-regulated in adipose and gingival tissues of high-fat diet-induced obese model mice

**Fig. 3:**
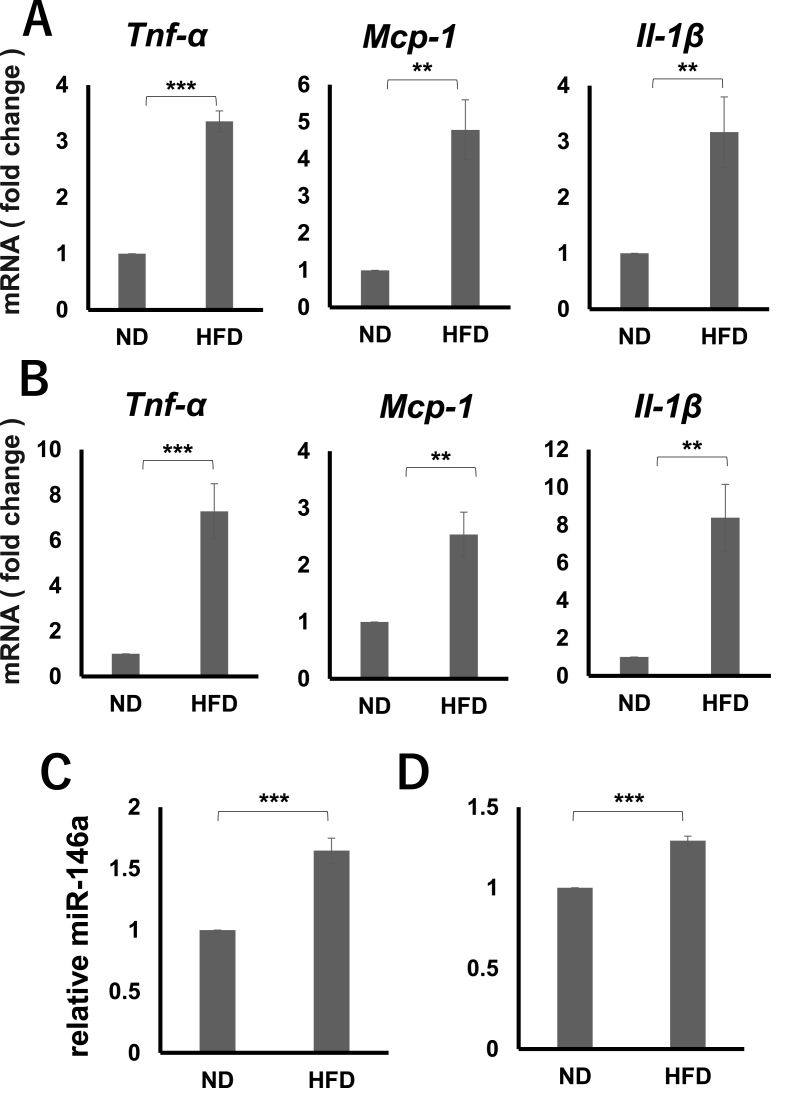
**Expression of inflammatory cytokine genes and miR-146a in adipose and periodontal tissues of high fat diet-induced obese mice and its control.** Real-time PCR analysis A, B) Expression of *Tnf-α*, *Il-6*, and *Mcp-1* in adipose(A), gingival(B) tissues from mice fed either a normal or a high-fat diet. C, D) Expression of miR-146a in adipose(C), gingival(D) tissues from mice fed either a normal or a high-fat diet. **P < 0.01, ***P < 0.001 (n = 3), Student's *t-*test.

When the test mice were fed on a high-fat diet for 9 weeks (i.e., starting when mice were 4 weeks old till they were of 12 weeks of age), inflammatory genes expression in adipose tissue was significantly up-regulated as compared to those of normal-diet fed mice (Fig. 3A). Similar results were observed in the gingival tissues of high-fat diet fed mice (Fig. 3B). Additionally, miR-146a expression in both adipose and gingival tissues, were significantly up-regulated in high-fat fed mice as compared to the normal-diet fed mice (Fig. 3C and D).

### 3.4 Continuous miR-146a administration protects mice from developing high-fat diet-induced obesity

**Fig. 4:**
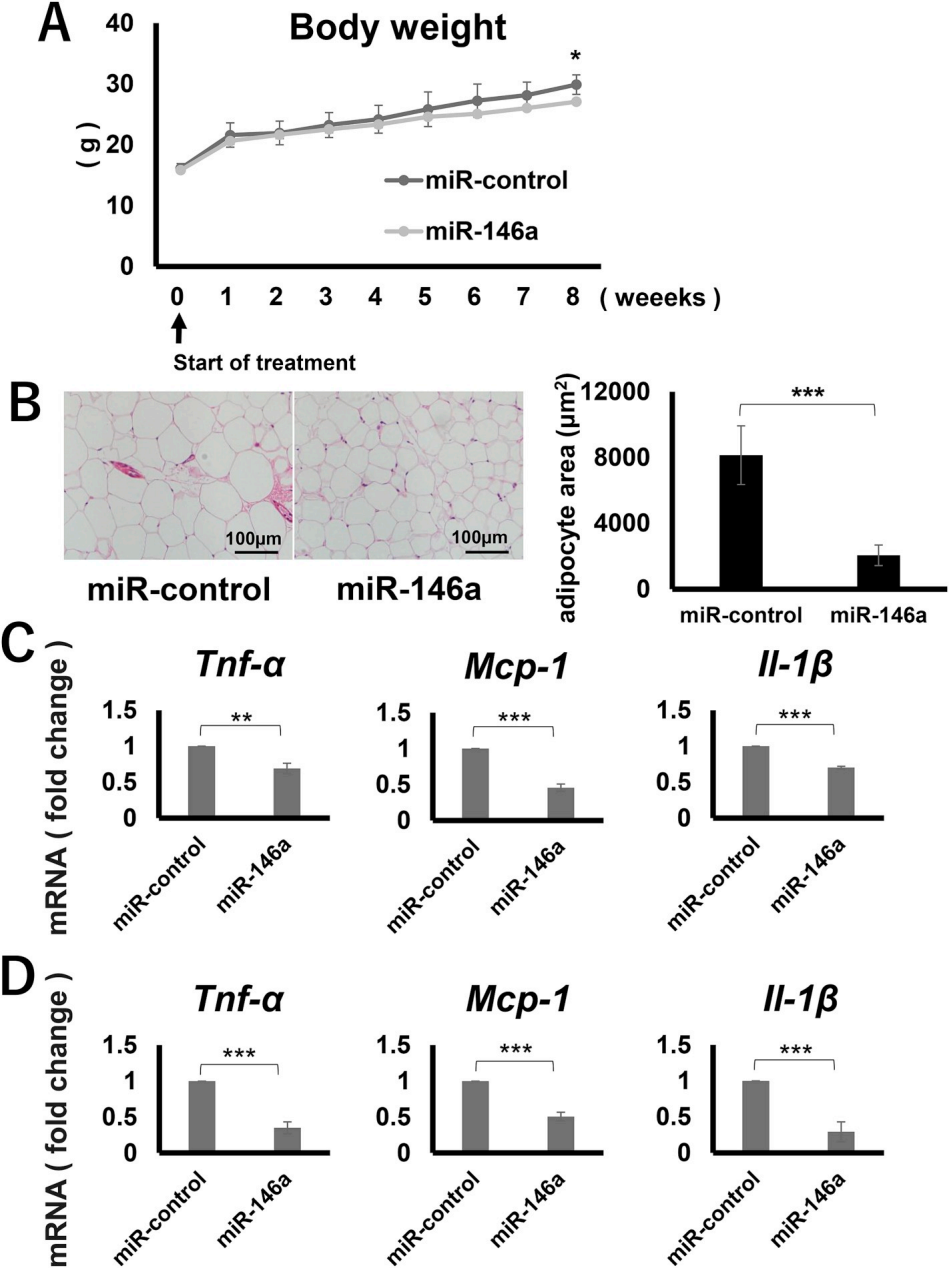
The effects of continuous injection of miR-146a on high-fat diet-induced obesity. A) Changes in body weight was monitored. B) HE staining of epididymal adipose tissues in each test group. The average adipocyte size of each group. Cell size was analyzed using Image J. C, D) Expression of *Tnf-α*, *Il-6*, and *Mcp-1* in adipose(C), gingival(D) tissues from each group as quantified by real-time PCR. *P < 0.05, **P < 0.01, ***P < 0.001 (n = 3), Student's *t*-test.

Since, miR-146a expression was up-regulated in high-fat diet-induced obese mice, we hypothesized that miR-146a might suppress the inflammatory responses by negative feedback mechanisms and further wanted to understand it's *in vivo* effects. It was observed that the high-fat diet fed mice, which were injected with miR-146a, once a week for 9 weeks via tail vein, were protected from diet-induced obesity at 12 weeks of age in comparison to the control miRNA-injected, high-fat diet fed mice (Fig. 4A). Additionally, adipocyte size was significantly suppressed in miR-146a-infused mice as compared to the control (Fig. 4B). Adipose tissue expression of the inflammatory cytokine genes was significantly down-regulated in miR-146a-treated mice (12 weeks old) (Fig. 4C). Similar effects were observed in the gingival tissues as well (Fig. 4D).

## 4 Discussion

miR-146a was noted to be expressed in the inflamed adipose and periodontal tissues, and also in the adipocytes and gingival fibroblasts, by macrophage-derived supernatants under inflammatory stimuli. miR-146a transfection resulted in decreased inflammatory cytokine gene expression in both, adipocytes and fibroblasts. miR-146a has been reported to suppress IRAK1 or TRAF6, expressions, both of which are signaling molecules, known to mediate TNF-α signals. In this study, on transfecting miR-146a into macrophages, macrophage derived TNF-α production was observed as significantly suppressed. We have previously reported that anti-TNF (a neutralizing antibody) markedly suppressed IL-6 and MCP-1 production in the adipocytes co-cultured with macrophages, in the presence of LPS, and suggested that macrophage-derived TNF-α plays an important role in up-regulating these cytokines [15]. Therefore, miR-146a was speculated to suppress macrophage-derived TNF-α production and the subsequent IL-6 and MCP-1 production in the adipocytes and gingival fibroblasts. Furthermore, in chronic adipose tissue inflammation, T-lymphocytes recruitment by macrophages is reported to play an important role in sustained inflammation [[16], [17], [18]]. Interestingly, miR-146a has also been reported to suppress ccr7 expression in the T-cells [19,20]. We have previously reported that ccr7 null mice were protected from diet-induced obesity and adipose tissue inflammation [21], and revealed an increase in brown adipocyte markers in the white adipose tissues. Furthermore, increased energy expenditure was also noted together with an increase in brown adipocyte activation markers expression [22]. We also confirmed ccr7 down-regulation in miR-146a-treated mice via tail vein (data not shown). Therefore, it could be possible that miR-146a not only regulates innate immune responses, but also down-regulates the subsequent T-cell recruitment into the inflamed tissues.

Two homologues of miR-146 (miR-146a, miR-146b) are known to exist. miR-146a is located on chromosome 5, while miR-146b on chromosome 10. In mice, miR-146a is located on chromosome 11, whereas, miR-146b on chromosome 19. Both miR-146a and miR-146b, share identical functions and their representative target molecules are reported to be IRAK1 and TRAF6 [23]. IRAK1 phosphorylation, hence activation, results in this molecule's binding with TRAF6, which activates the MAP kinase (JNK, p38 MAPK) and NF-κB pathways [24]. In this study, miR-146a transfection also resulted in IRAK1 and TRAF6 down-regulation and reduced JNK phosphorylation. Therefore, miR-146a appeared to play an important role in the regulation of inflammatory responses.

miR-146a has been reported to be associated with endotoxin tolerance in monocytes. Sustained stimulation of monocytes for several days with low endotoxin level results in miR-146 induction and also suppresses the inflammatory response against higher concentration of endotoxin stimulation [25]. miR-146a has been reported to suppress the endotoxin-induced inflammatory responses in both cultured monocytes and mice models [26,27]. Additionally, miR-146a null mice have been documented to exhibit enhanced sensitivity towards bacterial endotoxin and increased cytokine productivity; and towards fatal endotoxin shock [28]. miR-146a over-expression is also reported to suppress foam cell formation from macrophage and related cytokine production [29]. With continuous miR-146a injection, we observed down-regulated adipose and gingival tissue inflammatory responses *in vivo*. Therefore, miR-146a-mediated feedback loop might be effective in long-term immune regulation in inflammation-related diseases such as obesity and periodontal disease.

Although, extracellular miRNAs are known to enter the cells and suppress target genes expression [30], however, plasma contains massive RNase amounts, therefore, the artificially injected miRNAs are immediately lysed [31]. To overcome this problem, commercially available transfection reagent was utilized in the present study, via tail vein to note miR-146a effects on the chronic inflammatory conditions. The effects are also required to be confirmed with oral administration, in our future study. For that purpose, exosome or micro vesicle-mediated administration has been suggested [32]. Mast cell-derived exosome containing miRNAs have been reported to be functionally transferred into recipient mast cells [33]. We have previously reported that when mice were fed on epicatechin (major component of cocoa flavonoid) mixed diet, it resulted in protection of these mice from developing diet-induced obesity and subsequent insulin resistance [34]. It is also reported that ingested nutritional component itself contained some detectable amounts of miRNAs in the circulation [35], and, therefore, it is possible that target miRNAs could be combined with functional foods.

## Funding

This work was supported by Japan Society for the Promotion of Science (JSPS) KAKENHI, Japan. Grant Numbers; JP18K17071, JP16H05555, JP18H06328.

## Declaration of competing interest

The authors declare that none of the authors have potential conflict of interest.

## Appendix A Supplementary data

Fig. S1 Real-time PCR analysis (expression of miR-146a). A) miR-146a transfected 3T3-L1 adipocytes co-cultured with macrophages. B) miR-146a transfected ESK-1 cells co-cultured with macrophages. C) miR-146a transfected macrophages. Fig. S1
