# Supplementary figures and images for: Anti-inflammatory effects of miRNA-146a induced in adipose and periodontal tissues
Source: Biochem Biophys Rep. 2020 Apr 21;22:100757. doi: 10.1016/j.bbrep.2020.100757 (PMC7178317; doi:10.1016/j.bbrep.2020.100757)

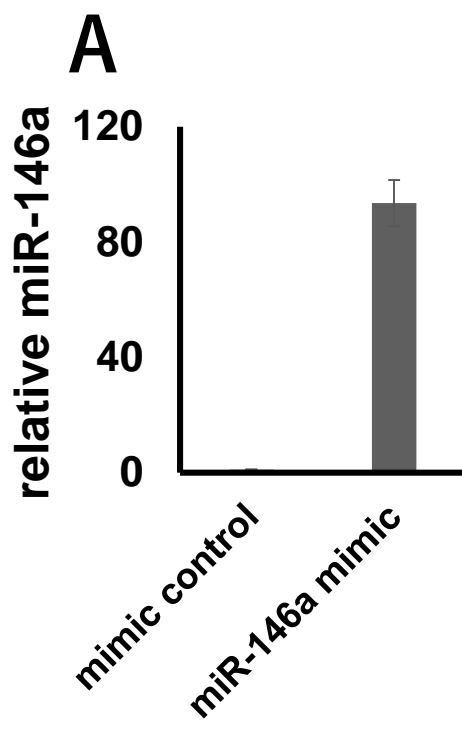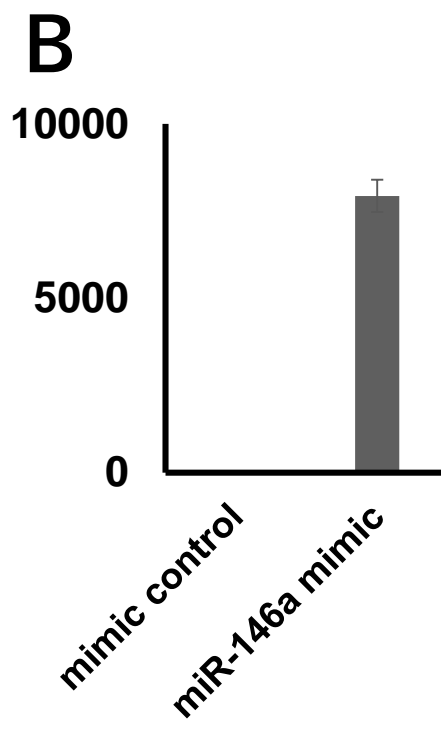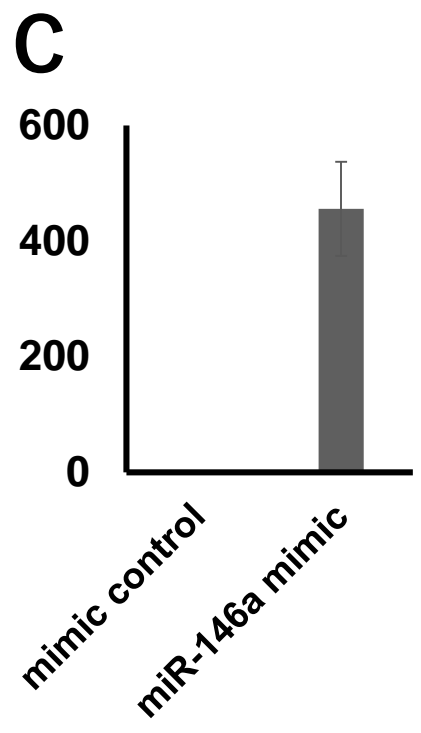

Supplement: Fig. S1 — Real-time PCR analysis (expression of miR-146a). A) miR-146a transfected 3T3-L1 adipocytes co-cultured with macrophages. B) miR-146a transfected ESK-1 cells co-cultured with macrophages. C) miR-146a transfected macrophages. [file mmc1.pdf]
